# Histopathological analysis for human articular cartilage using a Raman probe system

**DOI:** 10.1117/1.JBO.31.9.097005

**Published:** 2026-09-29

**Authors:** Hiroshi Kiyomatsu, Kosuke Toda, Takashi Katagiri, Akinori Taketani, Kaori Sugiyama, Tatsuhiko Kutsuna, Tomofumi Kinoshita, Takashi Tsuda, Masaki Takao, Yusuke Oshima

**Affiliations:** a Ehime University Graduate School of Medicine, Department of Orthopaedic Surgery, Ehime, Japan; b University of Toyama, Graduate School of Pharma-Medical Sciences, Toyama, Japan; c University of Toyama, Research Center for Pre-Disease Science, Toyama, Japan; d University of Toyama, Faculty of Engineering, Toyama, Japan; e National Defense Medical College Research Institute, Division of Bioinformation and Therapeutic Systems, Saitama, Japan; f Oita University, Department of Gastroenterological and Pediatric Surgery, Faculty of Medicine, Oita, Japan

**Keywords:** raman spectroscopy, osteoarthritis, joint cartilage, raman probe

## Abstract

**Significance:**

The molecular pathogenesis of degenerative alterations in articular cartilage is still not fully understood. Raman spectroscopy using a fiber-optic probe system is a promising approach for the early diagnosis and prevention of joint disorders such as osteoarthritis.

**Aim:**

This study aims to characterize the degenerative changes in the joint articular cartilage using a hollow-core fiber Raman probe designed for future arthroscopic use.

**Approach:**

Human articular cartilage specimens were obtained from 12 patients who underwent knee (n=5) or shoulder (n=7) arthroplasty. All surgical specimens were measured ex vivo after fixation in 10% neutral buffered formalin. The articular cartilage tissues at the knee, hip, and shoulder joints were obtained from a single cadaver donor. Raman spectra of the cartilage specimens were acquired and analyzed using a handheld Raman probe system.

**Results:**

The hollow-core fiber Raman probe detected molecular differences between less-degenerated and degenerated areas in human shoulder and knee articular cartilage *ex vivo*, with clearer separation in the knee, and additionally revealed joint-specific baseline spectral features among cadaveric shoulder, hip, and knee cartilage.

**Conclusions:**

These results support the novelty and translational potential of this probe as a future *in vivo* arthroscopic tool for objective molecular assessment of cartilage degeneration.

## 1 Introduction

Osteoarthritis (OA) is a representative degenerative joint disease in middle-aged and older adults and markedly impairs quality of life and increases medical and long-term care burden through pain and functional disability.^1^^,^^2^ Recent epidemiological studies have shown that, with population aging and rising obesity rates, the prevalence of OA—particularly in the knee, hip, and hand—is increasing worldwide.^1^^,^^3^ OA is now recognized not merely as a disease of the articular cartilage but as a “joint organ disease” involving reciprocal interactions among multiple tissues, including subchondral bone, synovium, ligaments, and periarticular muscles and tendons.^3^^,^^4^ Accordingly, there is a growing demand for quantitative assessment of pathological changes across joint tissues from the early stages of disease.

Articular cartilage is a specialized extracellular matrix composed of ∼70% water and, in terms of dry weight, predominantly a type II collagen fibrillar network and proteoglycans.^5^ Disorganization of collagen fibril orientation and loss of proteoglycan content reduce the mechanical properties of cartilage and are considered to be pathological changes that arise from the early stages of OA progression.^3^^,^^5^^,^^6^ Although these changes can be visualized by histopathologic assessments such as hematoxylin–eosin and Safranin O staining, they require biopsy, and therefore it is not straightforward to perform repeated, real-time evaluation during minimally invasive surgery.

In clinical practice, the diagnosis and severity grading of OA still rely heavily on the assessment of joint space narrowing and osteophyte formation on plain radiographs together with patient-reported pain and functional limitation.^1^^,^^2^ MRI and ultrasonography can depict soft-tissue and bone marrow lesions, and techniques such as optical coherence tomography (OCT), polarization-sensitive OCT (PS-OCT), and arthroscopic indentation have been applied to evaluate the microstructure and biomechanical properties of cartilage.^7^^–^^9^ However, these modalities primarily capture morphological and structural–mechanical alterations and do not directly reflect changes in molecular composition.^6^^,^^7^ Using nonlinear optical approaches, we have also reported that quantitative analysis of collagen second-harmonic generation (SHG) signals obtained by two-photon excitation microscopy is useful for detecting early alterations of the cartilage collagen network in animal models of OA.^10^

Raman spectroscopy is a spectroscopic technique that analyzes inelastic scattering (Raman scattering) of laser light incident on a sample to obtain label-free and nondestructive information about molecular species and their chemical bonding states.^6^^,^^11^ It is therefore well suited for biochemical evaluation of collagen- and proteoglycan-rich tissues such as cartilage. In cartilage Raman spectra, peaks are observed at $856\;{\mathrm{cm}}^{-1}$ (proline and other collagen-related modes), $936\;{\mathrm{cm}}^{-1}$ (C─C), $1250\;{\mathrm{cm}}^{-1}$ (amide III), $1448\;{\mathrm{cm}}^{-1}$ (${\mathrm{CH}}_{2}$ bending), and $1659\;{\mathrm{cm}}^{-1}$ (amide I) as collagen-derived bands, together with a proteoglycan-derived band at 936 (C─O─C stretching) and $1061\;{\mathrm{cm}}^{-1}$ attributed to sulfate groups ($O─{\mathrm{SO}}_{3^{-}}$). These features reflect alterations in matrix composition.^6^^,^^11^ Indeed, a growing body of work includes reviews of Raman spectroscopy in bone and joint disorders^11^ and reports on its application to OA diagnosis and grading using human synovial fluid and articular cartilage samples.^12^^,^^13^

In recent years, Raman spectroscopy of articular cartilage has progressed from ex vivo tissue analysis using confocal Raman microscopy to *in vivo* and intraoperative applications employing fiber-optic probes.^6^^,^^11^^–^^13^ In particular, to achieve objective and quantitative assessment of cartilage biochemical status during arthroscopic surgery, it is essential both to establish spectroscopic–histopathologic correlations in human OA joints and to evaluate the performance of fiber-optic Raman probes that can be introduced through the arthroscope.

In this study, we used a hollow-core fiber Raman probe designed for future arthroscopic use to perform *ex vivo* measurements of human articular cartilage. The study addressed three sequential questions: (1) whether Raman spectra obtained from surgical shoulder specimens can distinguish less-degenerated and degenerated areas, (2) whether the same approach can detect differences between less-degenerated and degenerated areas in knee surgical specimens, and (3) whether baseline Raman profiles differ among shoulder, hip, and knee cartilage in cadaveric samples from a single donor. Through this design, we aimed to establish the spectroscopic basis for future in vivo molecular assessment of cartilage during arthroscopic surgery.

## 2 Materials and Methods

### 2.1 Patients and Surgical Specimens

Surgical specimens were obtained from 12 patients who underwent knee (n=5) or shoulder (n=7) arthroplasty in the Department of Orthopaedic Surgery, Ehime University Hospital, Toon, Ehime, Japan. All the knee articular cartilage specimens (distal femur) were obtained from osteoarthritis patients (n=5). For the shoulder joint, humeral heads were obtained from patients with osteoarthritis (n=1), proximal humeral fracture (n=1), or rotator cuff tear arthropathy (n=5). The resected specimens were immediately fixed with 10% neutral buffered formalin and stored until Raman spectroscopic measurement. All Raman measurements of the surgical specimens were performed ex vivo after formalin fixation. Written informed consent was obtained from all the patients who joined this study. The research design and the experimental protocol were approved by the Ehime University Hospital institutional review board (approval ID: 2305018).

### 2.2 Cadaver Samples

The cadaver samples were provided by the Division of Anatomy and Embryology, Ehime University Graduate School of Medicine. The articular cartilage tissues, including full-thickness cartilage layer and subchondral bone layer, were collected using a biopsy trepan 10 mm in diameter at the knee, hip, and shoulder joints, respectively, from a single cadaver donor. Use of donated cadaveric material was conducted under institutional procedures based on prior donor consent and relevant regulations (approval ID: 2305018).

### 2.3 Handheld Raman Spectroscopy System

Raman spectroscopic measurements of the articular cartilage tissues obtained from the patients and the cadaver were carried out using a hand-held Raman system equipped with a ball-lens hollow-fiber Raman probe (BHRP). We constructed a portable Raman spectroscopic system consisting of a handheld Raman probe (INT900-1, INTROTEC, Kawasaki, Japan), an excitation laser source (08-NLD(M) 785 nm, Cobolt, Solna, Sweden), and a spectrometer (SP2150, Teledyne Princeton Instruments, Trenton, New Jersey, United States) with a CCD detector (iVac-316, Andor, Belfast, United Kingdom). The BHRP is attached to the distal end of the handheld Raman probe unit. The specifications and working principles of the BHRP were previously described elsewhere.^14^ In brief, the BHRP was fabricated by gluing a 1 mm-diameter sapphire ball lens (43-638, Edmund Optics, Barrington, New Jersey, United States) to the tip end of a silver-coated glass capillary. The BHRP is flexible due to its external polymer coating. The back focal length (BFL) of the ball lens was ∼80  μm. Despite this focal position, preliminary experimental evaluation of the probe indicated that Raman signals could be detected from depths of up to ∼500  μm. The length of the hollow optical fiber is ∼150  mm, which is suitable for intraoperative use during arthroscopic surgery. The probe is housed in a stainless-steel protective sheath, which enables safe handling and prevents fiber breakage during arthroscopic procedures. The probe tip can be sterilized using standard clinical sterilization methods, including gamma irradiation and low-temperature gas sterilization. The average laser output power from the end of the fiber probe was ∼340  mW. No visible change in the cartilage surface was observed immediately after the laser irradiation, and no measurement-related adverse events were identified histopathologically.

### 2.4 Raman Spectral Data Acquisition

For Raman measurements of surgical specimens, two locations—the “less-degenerated area” and the “degenerated area”—were selected on the weight-bearing surface of the knee and shoulder joints based on macroscopic findings. All the specimens were rinsed with saline prior to Raman measurement to wash out the fixative reagents. Raman spectra were acquired with the probe tip placed in contact with the cartilage surface. The contact force and probe angle were adjusted to maximize the signal-to-noise ratio (SNR) of the measured Raman spectrum. For each case, a total of 30 spectra were obtained from the “less-degenerated area” and the “degenerated area,” respectively. The acquisition time was 0.5 s with three accumulations for each measurement. The cadaver specimen group comprised bilateral shoulder joints (humeral heads), bilateral hip joints (femoral heads), and both knee joints (femoral condyles) from the same individual. For each joint, Raman spectra were acquired from a total of 12 locations from regions with preserved macroscopic morphology in the same manner.

### 2.5 Preprocessing of Raman Spectral Data

Data processing and analyses were performed using MATLAB software (MathWorks, Natick, Massachusetts, United States). The raw Raman spectral data were preprocessed prior to exploratory analysis and quantitative analysis. Although the spectral resolution and the wavenumber sampling interval were $∼1\;{\mathrm{cm}}^{-1}$, the raw spectra were not evenly spaced along the Raman shift axis. Therefore, data interpolation was performed to obtain an evenly spaced Raman shift axis, repositioning each spectrum using linear interpolation at $1\;{\mathrm{cm}}^{-1}$ intervals. Second, to eliminate the periodic intensity fluctuations that occur across the entire spectrum due to wavelength dependence of the optical system and detector, as well as the etalon effect caused by multiple reflections within the optical system, the spectral data set was corrected by dividing the interpolated sample spectrum by the white light spectrum. Specifically, the white light spectrum was smoothed by applying a moving average filter with a window size of 11 points to reduce fine intensity fluctuations. Third, the oxygen Raman peak was eliminated, because Raman peaks originating from oxygen within the hollow optical fiber tend to be relatively prominent. We prespecified the wavenumber range of 1540 to $1565\;{\mathrm{cm}}^{-1}$ where the oxygen peak is expected to appear and applied a moving average filter to the spectral intensity in this region. The moving average processing converted the sharp oxygen peak into a smoother variation, reducing the impact of the oxygen peak while maintaining the continuity of the spectrum. For baseline correction, a polynomial approximation was applied to the spectral data within the target wavenumber range. The resulting approximation curve was subtracted as the baseline. The quadratic polynomial used for fitting was chosen to adequately represent the background shape while avoiding overfitting. This process removed background components, yielding a spectrum with enhanced Raman peak components. The smoothing processing was performed to stabilize the spectral analysis. The moving average method was applied again with a window size of 5 points. This processing reduced high-frequency components without significantly degrading peak shapes, yielding a spectrum suitable for analysis. Finally, data normalization was performed by dividing the smoothed spectra by their average intensity. This process aligned the overall intensity scale of the spectra, enabling analysis based on peak shape and relative intensity distribution.

### 2.6 Spectral Feature Extraction and Statistical Analysis

We performed exploratory analysis using principal component analysis (PCA) to compare spectra between less-degenerated and degenerated areas and among joints in both surgical specimens and cadaver samples. In addition, we conducted peak-based quantitative analyses of selected Raman bands. Integrated peak areas were extracted using the full width at half maximum (FWHM) of each peak within the preprocessed spectrum (820 to $1720\;{\mathrm{cm}}^{-1}$) at $856\;{\mathrm{cm}}^{-1}$ (proline), $920\;{\mathrm{cm}}^{-1}$ (C─C), $936\;{\mathrm{cm}}^{-1}$ (C─C/C─O─C), $1004\;{\mathrm{cm}}^{-1}$ (phenylalanine), $1061\;{\mathrm{cm}}^{-1}$ ($O─{\mathrm{SO}}_{3}^{\text{-}}$), $1250\;{\mathrm{cm}}^{-1}$ (amide III), $1379\;{\mathrm{cm}}^{-1}$ (${\mathrm{CH}}_{3}$), $1448\;{\mathrm{cm}}^{-1}$ (${\mathrm{CH}}_{2}$ bending), and $1659\;{\mathrm{cm}}^{-1}$ (amide I). The Mann–Whitney U test was used to assess the statistical significance of differences in relative Raman peak intensities. Statistical analyses were performed using non-parametric methods due to the limited sample size and nonnormal distribution of the spectral data. All tests were conducted as two-tailed tests, and results were interpreted at a significance level of p<0.05. For the surgical samples, Raman spectra were treated as nested data within each patient, and patient-level paired analyses were performed to ensure that spectra obtained from the same patient or region were not treated as independent biological replicates.

### 2.7 Histopathological Examination

All the specimens were evaluated histopathologically by cryo-sectioning followed by hematoxylin-eosin (HE) staining and Safranin O staining. In HE-stained tissue sections, nuclear morphology, cartilage layer structure (superficial, intermediate, and deep layers), fibrosis, and changes in cell density were evaluated. For Safranin O staining, staining intensity was used as an indicator of proteoglycan content, and the average intensity from the superficial to intermediate layers was assessed microscopically. For each region, specimens exhibiting surface smoothness, preserved matrix structure, and strong Safranin O staining were classified as “histologically normal.” Those showing surface roughening, fibrosis, disrupted cell arrangement, and reduced Safranin O staining were classified as “histological lesions.” These findings were compared with Raman analysis results.

## 3 Results

Spectral overview (Fig. 1). Raman spectra acquired from macroscopically defined less-degenerated and degenerated areas of surgical shoulder and knee specimens exhibited the characteristic bands of articular cartilage, including peaks at $856\;{\mathrm{cm}}^{-1}$ (proline), $920\;{\mathrm{cm}}^{-1}$ (C─C), $936\;{\mathrm{cm}}^{-1}$ (C─C/C─O─C), $1004\;{\mathrm{cm}}^{-1}$ (phenylalanine), $1061\;{\mathrm{cm}}^{-1}$ ($O–{\mathrm{SO}}_{3}$), $1250\;{\mathrm{cm}}^{-1}$ (amide III), $1379\;{\mathrm{cm}}^{-1}$ (${\mathrm{CH}}_{3}$), $1448\;{\mathrm{cm}}^{-1}$ (${\mathrm{CH}}_{2}$), and $1659\;{\mathrm{cm}}^{-1}$ (amide I) [Figs. 1(a)–1(d)]. Overlaid mean spectra for each joint showed similar overall profiles between less-degenerated and degenerated areas, with group differences reflected as changes in relative band intensities [Figs. 1(c) and 1(d)].

**Fig. 1:**
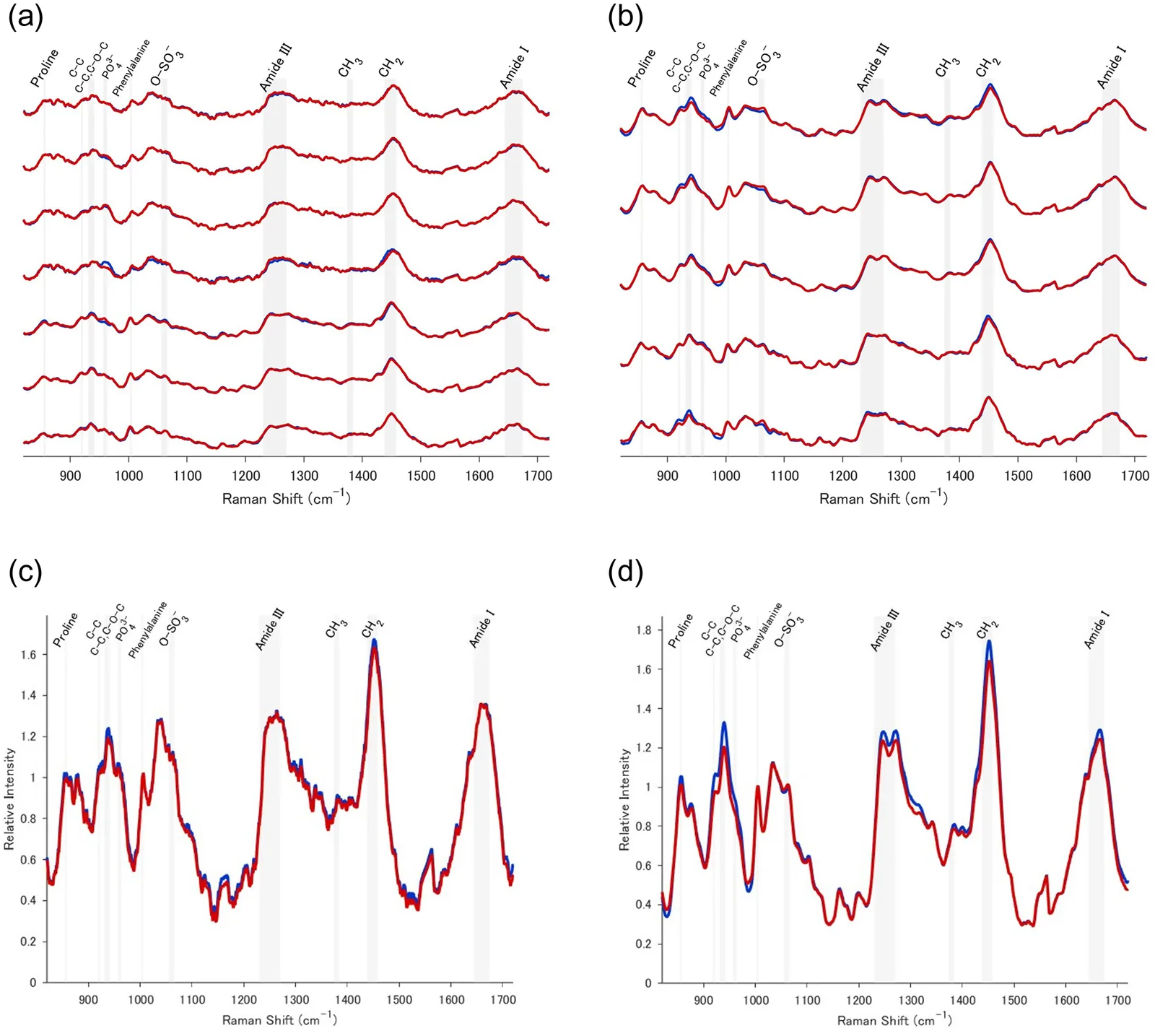
Averaged Raman spectra obtained from (a) shoulder joints and (b) knee joints for each patient. Averaged Raman spectra of (c) shoulder and (d) knee cartilage from all patients. Blue and red lines indicate the less-degenerated and degenerated areas, respectively.

To evaluate spectral characteristics associated with cartilage degeneration, principal component analysis was performed (Fig. 2). PCA score plots showed partial separation between spectra from the less-degenerated and degenerated areas in the shoulder dataset, most evident in the PC6–PC7 space [Fig. 2(a)]. In the knee dataset, separation between spectra from the less-degenerated and degenerated areas was observed in the PC2–PC4 space [Fig. 2(c)]. The corresponding loading plots showed contributions from major cartilage bands and from a band near $960\;{\mathrm{cm}}^{-1}$ [Figs. 2(b) and 2(d)].

**Fig. 2:**
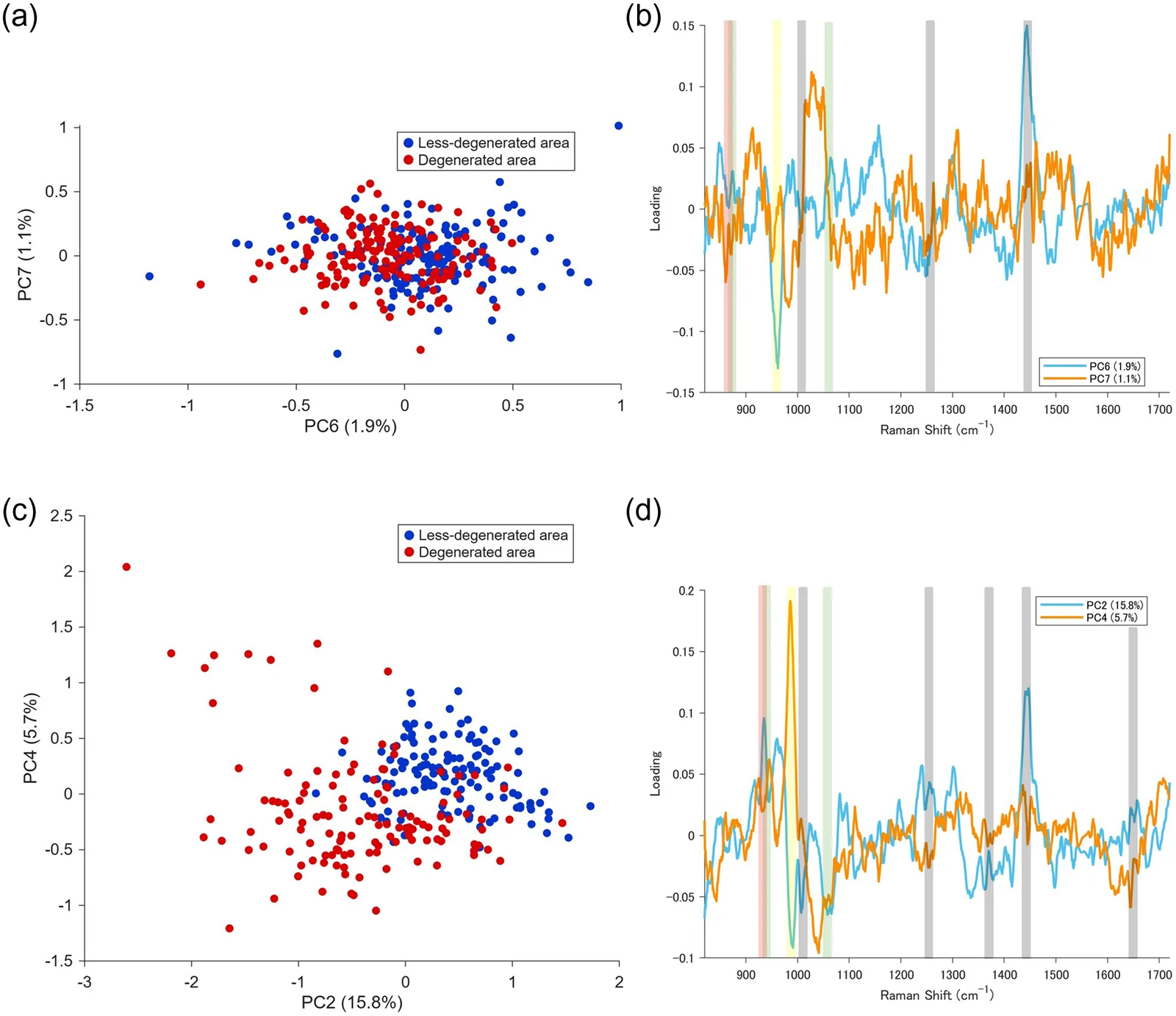
PCA score plots of all measured spectra and the corresopiding loading spectra for (a)–(b) shoulder joints and (c)–(d) knee joints from all patients. Major peaks corrosponding to Raman signal originated from collagen, proteoglycan and proteins are highlighted in red, green and gray, respectively. The peak around $960\;{\mathrm{cm}}^{-1}$, derived from phosphate in subcohondral bone, is indicated with a yellow bar.

Peak-based comparison of the Raman spectra was performed as nested data within each patient. (Fig. 3, Table 1). In shoulder specimens, degenerated areas showed significantly lower normalized intensities at the $936\;{\mathrm{cm}}^{-1}$ (C─C in collagen/C─O─C in proteoglycan) compared with the less-degenerated areas (p<0.05), whereas differences at the other bands were not significant. In knee specimens, degenerated areas showed significantly lower values at 920 (C─C in collagen) and $936\;{\mathrm{cm}}^{-1}$ (C─C in collagen/C─O─C in proteoglycan) (p<0.01), while the other bands were not significant.

**Fig. 3:**
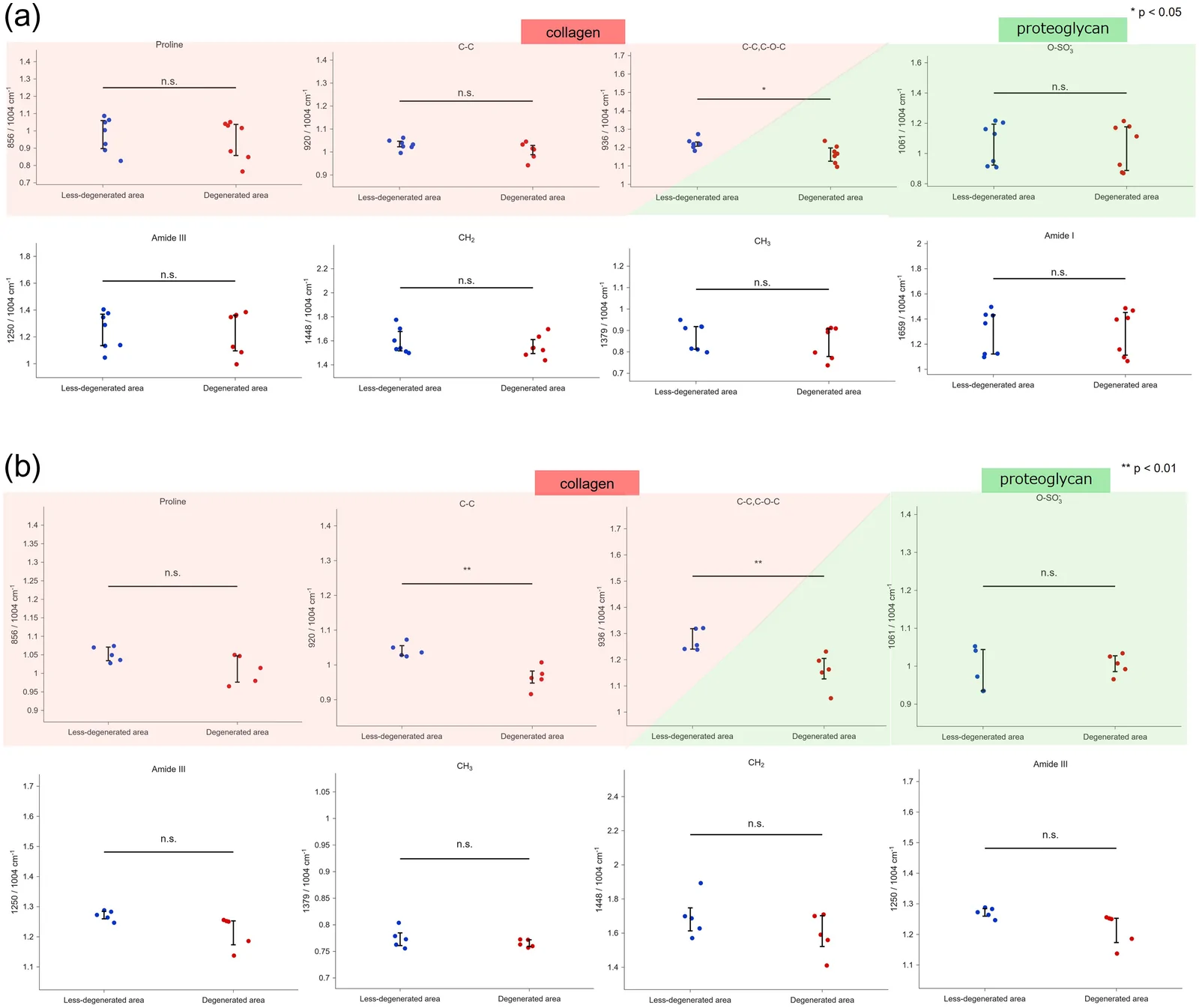
Comparison of relative Raman peak intensities between less-degenerated and degenerated areas in (a) shoulder and (b) knee joints for all patients. The peak around $936\;{\mathrm{cm}}^{-1}$ overlapped with both the C─C and the C─O─C vibrational modes originating from collagen and proteoglycan, respectively.^15^

**Table 1:** Summary of Raman spectral changes between shoulder and knee joints.

	Collagen		Proteoglycan		Protein			
	Proline	C─C	C─C, C─O─C	$O─{\mathrm{SO}}_{3}^{\text{-}}$	Amide III	${\mathrm{CH}}_{3}$	${\mathrm{CH}}_{2}$	Amide I
Shoulder	n.s.	n.s.	↓	n.s.	n.s.	n.s.	n.s.	n.s.
Knee	n.s.	↓	↓	n.s.	n.s.	n.s.	n.s.	n.s.

Degenerated-area values relative to the less-degenerated area (↓ Lower than the less-degenerated area).

Representative shoulder case [Fig. 4(a)]. In the representative shoulder specimen, the degenerated area showed surface irregularity and reduced Safranin O staining compared with the paired less-degenerated area [Fig. 4(b)]. PCA of Raman spectra from this specimen separated the less-degenerated and degenerated areas in the score plot [Fig. 4(c)]. The Raman peaks originating from the cartilage matrix, which contribute to the separation between the less degenerated and degenerated areas, were evident in the principal component loadings [Fig. 4(d)].

**Fig. 4:**
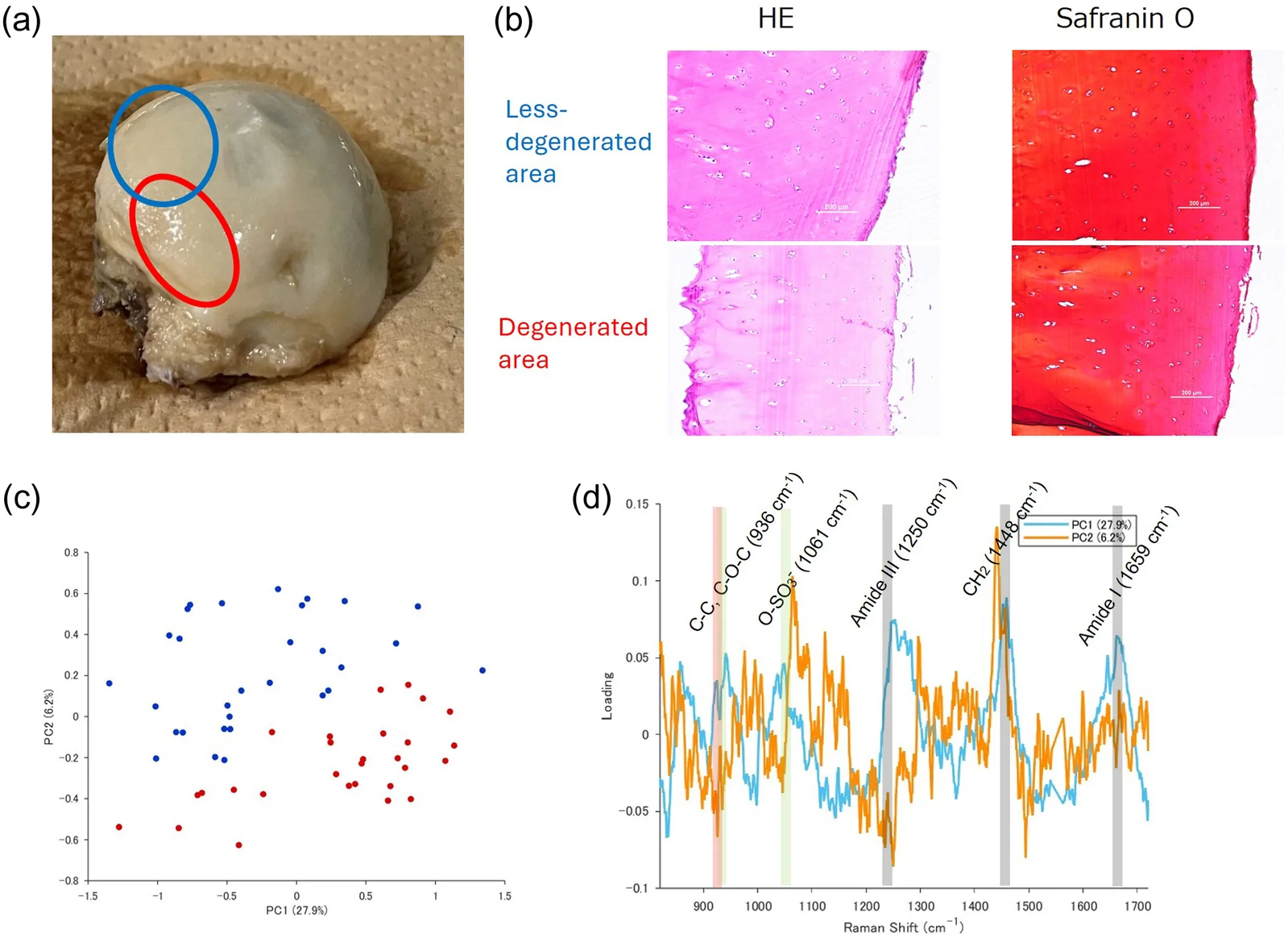
Representative specimens of shoulder joint cartilage (humeral head). (a) macroscopic photograph, (b) HE staining and Safranin O staining in tissue section, (c) PCA score plots (blue: less-degenerated area, red: degenerated area), and (d) PCA loading spectra (PC1 and PC2).

Representative knee case [Fig. 5(a)]. In the representative knee specimen, the degenerated area showed surface fibrillation/matrix disruption and reduced Safranin O staining compared with the paired less-degenerated area [Fig. 5(b)]. PCA of Raman spectra from this specimen separated the less-degenerated and degenerated areas in the score plot [Fig. 5(c)]. The Raman peaks originating from the cartilage matrix, which contribute to the separation between the less degenerated and degenerated areas, were evident in the principal component loadings [Fig. 5(d)].

**Fig. 5:**
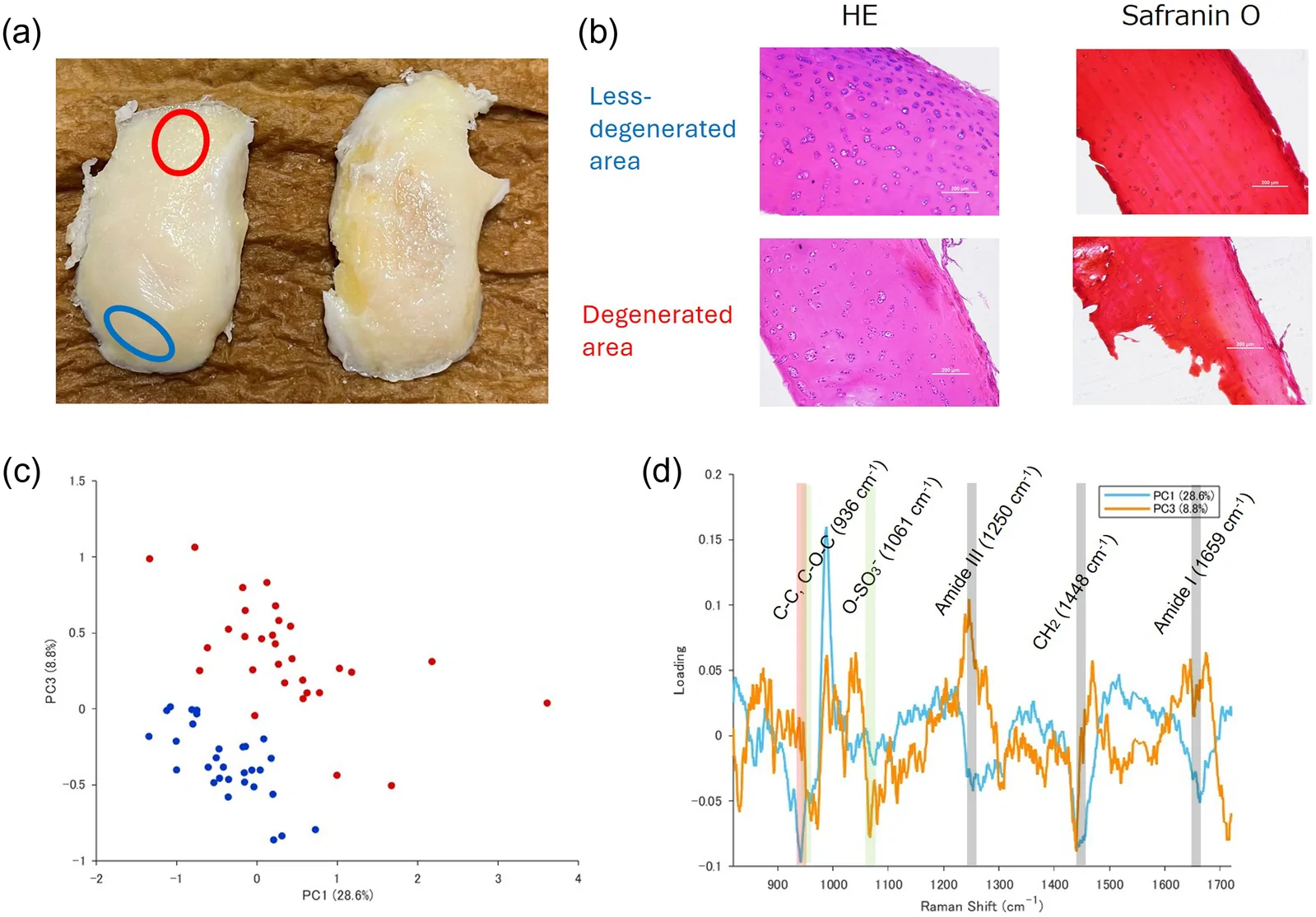
Representative specimens of knee joint cartilage (distal femur). (a) macroscopic photograph, (b) HE staining and Safranin O staining in tissue section, (c) PCA score plots (blue: less-degenerated area, red: degenerated area) and (d) PCA loading spectra (PC1 and PC2).

Cadaveric multijoint comparison [Fig. 6(a)]. In cadaveric cartilage from a single donor, Raman spectra from the shoulder, hip, and knee joints shared the major cartilage bands but differed in their relative intensities [Fig. 6(b)]. PCA clustered spectra by joint type, and peak-based analysis showed significant differences in selected band intensities among shoulder, hip, and knee cartilage [Fig. 6(c)].

## 4 Discussion

This study provides four key observations relevant to the development of an arthroscopy-compatible Raman probe for cartilage assessment. First, the hollow-core fiber probe captured reproducible Raman fingerprints from human articular cartilage *ex vivo*. Second, the probe detected molecular differences between less-degenerated and degenerated areas in both shoulder and knee surgical specimens. Third, the discrimination was markedly clearer in the knee than in the shoulder and was supported by corresponding histological differences in representative cases. Fourth, cadaveric measurements revealed joint-specific baseline spectra among shoulder, hip, and knee cartilage, indicating that diagnostic algorithms may need to be joint-specific rather than universal.

The main novelty of this study lies in combining a miniaturized hollow-core fiber probe intended for arthroscopic deployment with human ex vivo cartilage measurements from multiple joints. Previous Raman work in osteoarthritis has mainly used benchtop confocal systems or preclinical fiber-based platforms.^14^^–^^20^ By showing that a contact-type probe with a distal diameter of ∼1  mm can detect degeneration-related signatures in human surgical specimens, the present study advances Raman spectroscopy from laboratory tissue characterization toward a clinically deployable optical-biopsy approach.

The qualitative spectral comparison in Fig. 1 and the principal component analysis in Fig. 2 indicate that degeneration does not generate an entirely new spectral phenotype; rather, it changes the relative contribution of existing matrix bands. This is biologically plausible because osteoarthritis begins with matrix disorganization and compositional imbalance within the collagen–proteoglycan framework rather than complete replacement of tissue constituents.^3^^–^^6^ The fact that the knee score plot separated more clearly than the shoulder suggests that the magnitude and consistency of these molecular shifts are joint- and cohort-dependent.

The band-by-band analysis supports the interpretation that collagen-related changes dominate the Raman signature of degeneration in the present specimens. In both joints, decreases were observed in bands assigned to proline, C─C, C─C/C─O─C, ${\mathrm{CH}}_{2}$, and amide regions, and these changes were broader in the knee. Because the collagen fibrillar network is the principal load-bearing component of articular cartilage,^5^ disruption of its organization or local concentration is expected to alter these bands. This interpretation agrees with previous Raman studies linking collagen-related spectral changes to cartilage degeneration in human shoulder and hip samples.^15^^,^^17^

At the same time, the proteoglycan-related $O─{\mathrm{SO}}_{3^{-}}$ band did not consistently separate less-degenerated and degenerated areas despite the reduced Safranin O staining in the representative shoulder and knee cases. This discrepancy is informative rather than contradictory. Notably, the shoulder surgical cohort included rotator cuff tear arthropathy cases (associated with massive rotator cuff tears), in which articular cartilage degeneration may have been relatively mild in some regions, potentially attenuating proteoglycan-related spectral contrasts. Proteoglycan depletion may be spatially heterogeneous, depth-dependent, and patient-specific, whereas the contact probe integrates signals over a finite area and depth. Previous Raman mapping studies have likewise shown marked local and depth-dependent heterogeneity of glycosaminoglycan-related signals in native and degraded cartilage.^18^^,^^19^ In our approach, although the penetration depth is limited, multipoint acquisition across the cartilage surface allows spatially heterogeneous degenerative features to be reflected in Raman peak variability among patients. Therefore, degeneration is better captured by multivariate spectral patterns than by reliance on a single sulfate peak.

The presence of a phosphate-related contribution in the loading plots suggests that degeneration-related separation was influenced not only by the superficial cartilage matrix but also by the osteochondral background. The phosphate peak is not dominant because the cartilage thickness in human articular cartilage is ∼2 to 4 mm, and the Raman signal is surface weighted due to its limited penetration depth (<0.5  mm). In severe OA cases, thinning of the cartilage layer can lead to partial exposure of subchondral bone, in which case a phosphate peak may appear.^21^ This is consistent with the current concept of osteoarthritis as a whole-joint disease involving coordinated changes across cartilage, calcified cartilage, and subchondral bone.^3^^,^^4^ From a translational perspective, such mixed osteochondral information may be advantageous, because clinically relevant lesions rarely represent isolated superficial cartilage events.

The weaker and less consistent separation in the shoulder deserves particular attention. The shoulder surgical series was heterogeneous and included osteoarthritis, rotator cuff tear arthropathy, and proximal humeral fracture, whereas all knee surgical specimens were derived from osteoarthritic joints. In addition, rotator cuff tear arthropathy cases (often related to massive rotator cuff tears) may exhibit comparatively mild articular cartilage degeneration in some regions, which could dilute degeneration-associated Raman differences in pooled analyses. PCA is a variance-based method. Therefore, lower variance in the shoulder joint case within a dataset naturally results in greater overlap between “less degenerated” and “degenerated” areas. PCA is a variance-based method. Therefore, lower variance within the shoulder dataset naturally results in greater overlap between spectra from ‘less degenerated’ and ‘degenerated’ regions. The degree of clustering separation between joints reflects differences in dataset variance and should not be interpreted as a direct indicator of osteoarthritis severity. Clustering differences are attributable to dataset characteristics rather than histopathological severity. Raman spectroscopic analysis using the hollow-core fiber probe, despite its limited signal collection depth, provides pathological characteristics based on patient-specific spectral variance, offering a distinct and complementary assessment framework that differs from conventional histological grading. Moreover, the cadaver comparison in Fig. 6 showed that baseline Raman profiles differ among joints. Together, these findings suggest that diagnostic thresholds derived from one joint should not be transferred directly to another joint and that shoulder cartilage may require disease-specific and joint-specific reference datasets.

**Fig. 6:**
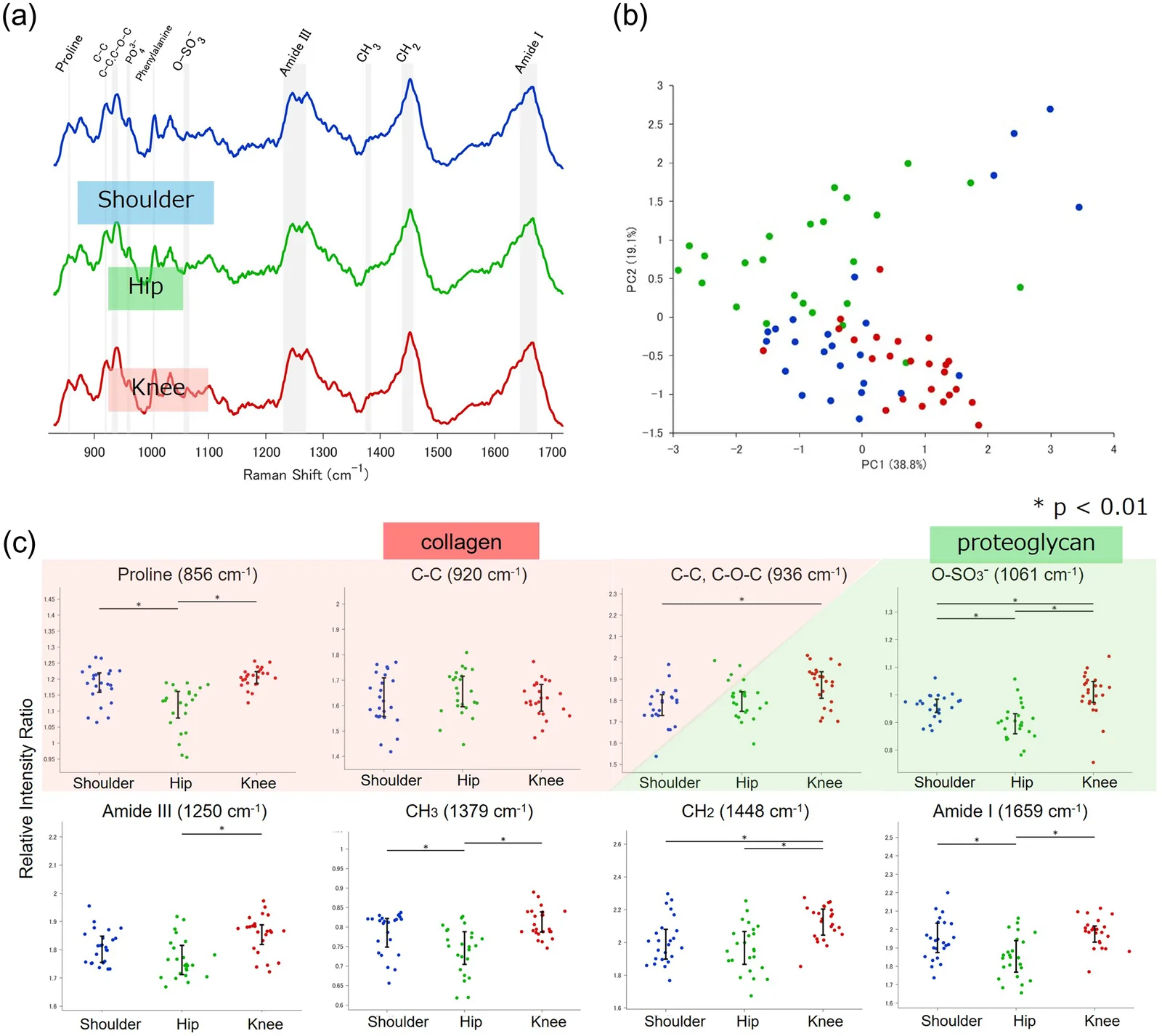
(a) Averaged Raman spectra obtained from the shoulder, hip, and knee joints of a cadaver, respectively. (b) PCA score plots (blue: shoulder, green: hip, red: knee). (c) Quantitative analysis of Raman peak intensities associated with cartilage matrix composition among joints in a cadaver.

These joint-specific findings represent an important developmental message for future in vivo use. A practical arthroscopic Raman system should ideally be trained with joint-matched data and should interpret spectra in the context of the local anatomical environment. In addition, because Raman output is numerical and high-dimensional, integration with machine-learning classifiers is a realistic next step. Recent reviews indicate that Raman-based osteoarthritis classification is promising but still limited by small cohorts and lack of external validation.^20^ The present study contributes a clinically relevant human dataset that can support such future modeling.

Several limitations should be acknowledged. Measurements were performed ex vivo using formalin-fixed specimens. Although formalin fixation alters the molecular and histological properties of the cartilage matrix, previous studies have shown that its impact on Raman-based evaluation of degenerative changes—such as collagen orientation—is limited.^22^ Fixative reagents may remain within the tissue; therefore, specimens must be thoroughly rinsed with saline prior to Raman spectroscopic measurements. Under fresh tissue or in vivo conditions, synovial fluid and blood may interfere with Raman measurements due to strong Raman signals originating from lipids and hemoglobin. Therefore, an appropriate washing procedure is essential for both *ex vivo* and *in vivo* measurements. In addition, the sample size was limited, the shoulder cohort was clinically heterogeneous, and the cadaver comparison was based on one donor. Region selection was based on macroscopic inspection rather than a formal arthroscopic grading atlas, and external validation in an independent cohort was not performed. Nevertheless, the consistent relationship between Raman findings, histological differences, and joint-dependent trends supports the biological validity of the observations.

## 5 Conclusion

In conclusion, the hollow-core fiber Raman probe detected molecular differences between less-degenerated and degenerated areas in human shoulder and knee articular cartilage *ex vivo*, with clearer separation in the knee, and additionally revealed joint-specific baseline spectral features among cadaveric shoulder, hip, and knee cartilage. These results support the novelty and translational potential of this probe as a future *in vivo* arthroscopic tool for objective molecular assessment of cartilage degeneration.

## Data Availability

The datasets generated and analyzed during the current study are not publicly available due to maintenance issues, but are available from the corresponding author on a reasonable request.
